# Late diagnosis of autism: exploring experiences of males diagnosed with autism in adulthood

**DOI:** 10.1007/s12144-022-03514-z

**Published:** 2022-08-03

**Authors:** Bomikazi M. Lupindo, Anastasia Maw, Nokuthula Shabalala

**Affiliations:** 1 University of Cape Town, Cape Town, South Africa; 2 Midrand, South Africa

**Keywords:** Autism spectrum disorders, High functioning, Males, Adulthood diagnosis

## Abstract

Autism Spectrum Disorder (ASD) is a pervasive neurodevelopmental disorder associated with qualitative impairments in social interaction, social communication and restricted, repetitive behaviour (American Psychiatric Association, 2013). Symptoms of ASD are first evident in infancy and childhood. However, individuals presenting with less overt ASD symptomatology may only be diagnosed in adulthood, when the expectation of independence and productivity results in a growing crisis for the individual. This study applied an exploratory qualitative research design to explore first-hand experiences of ten adult males (25 years and above) who were diagnosed with autism during their adulthood. Purposive sampling was used to select participants through the Neurodiversity Centre, Cape Town, South Africa. In-depth one-on-one interviews, guided by a semi-structured interview schedule were conducted. The thematic analysis technique and NVivo 12 qualitative analysis software were used to organise the data and identify themes. Three key themes emerged: failure to diagnose ASD in childhood despite signs and symptoms, ramifications of missed/misdiagnosis in childhood and adulthood on psychological well-being and the impact of receiving a diagnosis of ASD in adulthood. Missed/misdiagnosis had serious implications for psychological well-being throughout childhood and into adulthood. Late diagnosis resulted in missed opportunities for early intervention to address impairments associated with ASD. Receiving a diagnosis provided an explanation for long standing difficulties, offered a way forward in terms of developing coping strategies and allowed for self-acceptance. The implications of these findings for the development of better early screening and assessment for ASD are discussed and future research pathways suggested.

## Introduction

Autism Spectrum Disorder (ASD) is a pervasive neurodevelopmental disorder associated with atypical functioning and qualitative impairments in individuals across three major areas, namely, social interaction, social communication, and restricted, repetitive behaviour (American Psychiatric Association, 2013). In the past years, the global prevalence rate of ASD has increased significantly. The initial estimates were as low as 5 in 10 000 children (Lotter, 1966) and more recent studies indicate that 1 in 160 children globally have autism (World Health Organization, 2017). Although South Africa has no data on prevalence, there is no reason to believe that it is any lower than in global north countries, as the disorder has no boundaries with regards to ethnicity and socioeconomic status. The changes in the diagnostic criteria, better diagnostic tools, and the recognition of the spectrum nature of neurodevelopmental disorders, such as ASD, have contributed to improved diagnosis of autism (Silberman, 2015). This suggests that misdiagnosis/ missed diagnosis of autism is more likely to have occurred prior to this growing recognition, particularly so, for children and adolescents with “milder symptoms” or those regarded as being “high functioning”.^1^

ASD is often diagnosed during childhood, as symptoms indicative of autism tend to be evident during infancy and early childhood. However, in some instances, ASD is only detected at later stages in life for reasons such as individuals presenting with subtle symptoms, compensatory strategies used to camouflage ASD related challenges, as well as mistaking signs and symptoms of ASD for other psychiatric disorders (Bargiela et al., 2016; Wing & Potter, 2002). Furthermore, it has been argued that autism is a gender biased disorder; Baron-Cohen et al. (2005) describe it as a phenotype representation of the exaggerated form of the male brain, thus males who presented with milder symptoms of ASD may be missed/ misdiagnosed during childhood. Interestingly, research on late diagnosis of autism appears to have focused on the experiences of females (Bargiela et al., 2016; Lehnhardt et al., 2016). There is limited research on understanding the late diagnosis of autism in males and the impact this has had on them. In addition, Swierczynski (2018) highlights the need for cross-disciplinary linkages to create a unified body of knowledge that aims at better understanding ASD presentations and to aid the development of effective screening for and assessment of ASD and interventions for ASD. It is important to explore the impact of a late diagnosis and understand the factors which contribute to the late diagnosis of ASD, in both males and females. It is hoped that this information would contribute to more efficient screening for and assessment of ASD and the provision of support for the challenges ASD presents for developmental trajectories.

The support required for individuals diagnosed with ASD and their families comes at a high cost. In Australia, it is estimated that the median family cost of autism is $34 900 (AUD) per annum (Horlin et al., 2014). A further estimate of $1 400 (AUD) for the family per annum was reported as an increase in the cost of ASD for each additional symptom that individuals presented with. In the South African context, where the majority of the population relies on public health services (Statistics South Africa, 2017), access to diagnostic and treatment interventions may be further compromised as these services may not be available at public health care facilities. Early identification of ASD and supportive interventions offer the possibility of reducing the long-term costs associated with ASD for both individuals and their families, thus late diagnosis has significant financial and emotional ramifications. This further underscores the need for more efficient early screening, assessment and intervention.

Autism Spectrum Disorder has a profound impact on many aspects of life for individuals diagnosed with the disorder and their families. Individuals with conditions on the autism spectrum are at risk of difficulties related to social outcomes, occupational and economic issues that significantly impact their ability to live independently (DePape & Lindsay, 2016; Hendrickx, 2009, 2015). Furthermore, a diagnosis of ASD is associated with a higher risk of experiencing depression, anxiety, and suicidality (Leyfer et al., 2006), issues with conflicting identity (DePape & Lindsay, 2016; Lewis, 2016) and by extension families of individuals diagnosed with ASD are profoundly impacted by the disorder both emotionally and economically (Loukisas & Papoudi, 2016). These risk factors can be mitigated through timely diagnosis during the critical developmental period of childhood when available supports and interventions may affect life outcomes for diagnosed individuals (Hurlbutt & Chalmers, 2002). Furthermore, social, and environmental changes, such as those resulting from the COVID-19 pandemic, have been destabilizing and have had a psychological impact on individuals’ quality of life globally (Aki et al., 2020). These challenges have been found to be more profound within families of and individuals with ASD, necessitating intensified supportive measures to alleviate the challenges linked to ASD (Parenteau et al., 2020). Given the profound impact of ASD on individual functioning and families supporting those individuals, it is important to understand the factors that contribute to a late diagnosis to mitigate against this in the future, thereby reducing psychological distress and improving the quality of life for individuals and their families throughout the life span.

There is currently very limited research investigating the causes and impact of an adulthood diagnosis of autism. A total of five studies that focused on this area were found, namely, Bargiela et al. (2016), Bastiaansen et al. (2011), Davidocitch et al. (2015), Lehnhardt et al. (2016), and Lewis (2016). No studies were found within Sub-Saharan Africa. The aim of this study is to contribute to the growing literature within this subject area, specifically within low-income countries such as those found in Sub-Saharan Africa. The present study draws on previous studies, however, it aims to expand from a context that hasn’t been explored, particularly, the impact late diagnosis of ASD using a sample of male participants. Bargiela et al. (2016) provided a reference point for this study. Bargiela et al. (2016) focused on females, whilst this study is focused on males, to contributed to growing the literature and to allow for some comparison between men and women with a late diagnosis of ASD.

## Research questions

1. What factors contribute to delayed/ adulthood diagnosis^2^ of ASD in males, with specific references to signs and symptoms present in childhood?
2. What are the challenges experienced, prior and post-diagnosis, by males diagnosed with autism during adulthood and how have these challenges impacted their life trajectory?
3. What factors lead to seeking a diagnosis during adulthood?

## Method

### Study design and description of data collection site

This study applied an exploratory qualitative research design as a method of inquiry, as its aim was to understand how people socially construct meanings and perspectives. The study was conducted through the Neurodiversity Centre (located between Paarl, Stellenbosch and Franschhoek, in the Western Cape, South Africa). The Neurodiversity Centre (NDC) specializes in providing services for social communication challenges and autism spectrum disorders, thus, participants recruited for the study who met the inclusion criteria were easily accessible through the Centre. The sample size was small, nonparametric, and selected purposively as the study was interested in eliciting an in-depth qualitative experience as shared by the participants.

### Participants

The study aimed to recruit participants who were clients at the NDC and who were already seeking assistance for their ASD related challenges. The aim was to recruit 15 to 20 participants, however, given that autism is characterized by a qualitive impairment in social interactions and communication, the data being collected through one-on-one interviews limited the number of participants who were willing to engage in such a process. A total of 10 participants were willing to participate in the study. To be considered for participation, the participants had to have received a formal diagnosis of ASD after the age of 18 years. They had to be above the age of twenty-five as they would be considered as being fully fledged adults who have sought to engage in romantic and other significant social relationships, pursued work prospects, attempted living independently and experienced challenges that led to seeking help at NDC. In addition, participant selection was limited to those males who received their diagnosis six months (or longer) prior to this study being conducted. This was to ensure that participants had enough life experiences following diagnosis and, thus were able to provide rich data that was pertinent to the study, about their experiences before and after diagnosis.

### Sampling

The participants were sampled using the purposive sampling technique as it allowed the clinicians to select participants who could provide in-depth descriptions of their experiences and information that was pertinent to the study. Prospective participants who were identified as being suitable for the study, were approached by their clinicians at NDC as they had already established relationships with the clients and for confidentiality reasons. Once prospective participants had indicated a willingness to participate in the study, they were individually contacted by the training coordinator at the NDC to confirm their interest and to gain consent for the researcher to contact them. Those who confirmed their willingness to participate were then directly contacted by the researcher (the first author of this paper) inviting them to participate.

### Procedure

The study commenced immediately after receiving ethical clearance from the University of Cape Town (UCT) Human Research Ethics Committee. A formal letter requesting participation and outlining the aim of the study, along with the informed consent stating participants’ rights during the study, was sent (through the training coordinator at NDC) to those identified as prospective participants by their clinicians. Of the twenty participants who responded and showed an interest in participating in the study, ten participants were willing to come in for the interviews. Once participants had been recruited, data collection commenced. A schedule indicating the dates and times on which interviews could be conducted was distributed to the participants, allowing them to select their preferred interview appointment. The semi-structured interview schedule was then sent to each of the participants beforehand to allow them to familiarize themselves with the questions to reduce any uncertainty in relation to what would be asked of them in the interview. It also allowed the participants to consider and prepare their responses to ensure that they were satisfied with the responses they provided. It was hoped that receiving the schedule beforehand would reduce the anticipatory anxiety participants may have had regarding the interview and engaging with an unfamiliar person. Two interview venues were made available to accommodate participants who lived in Cape Town and those who lived in Stellenbosch; the venues were the UCT Child Guidance Clinic and the NDC respectively. To ensure that the participants were comfortable and to ease their anxiety about meeting the researcher for the first time, the first ten minutes of the interview was spent having general conversations with the participant. Once participants were settled, the researcher explained the study to them, went through the informed consent, and addressed any questions that emerged.

### Data collection

Life history interviews were conducted with the participants to help them reflect on their life experiences using their own words and timelines. While it is recognized that social communication is a challenge in autism, it was hoped that for those who were eager to participate and were willing to be interviewed, interviews would offer data that is more in-depth than what would be elicited through questionnaires. Furthermore, the participants were receiving counselling at NDC for their difficulties, therefore, it was hoped that the familiarity of in-person counselling would aid in their ability to participate and engage in interviews. An interview schedule was used as a guide to conduct the interviews, which were semi-structured in nature and involved the researcher asking each participant predetermined open-ended questions. The interview schedule used by Bargiela et al. (2016) was used as a guide to develop a semi-structured questionnaire informed by the objectives of the study. This study was used as a guide as Bargiela et al. (2016) conducted a similar study to the current research (albeit they investigated the female phenotype of women diagnosed with ASD in adulthood). Prior to the interviews, a role-play of an interview was conducted by the researcher and a clinician from the NDC to consider how the questions might be experienced by the participants and to prepare the interviewer in terms of how to best engage with participants during the interview. Any themes or information given in response to the open-ended questions served as a guideline for further probing by the researcher, at their own discretion as suggested by Neuman (2014). In cases where participants were highly anxious, interviews were rescheduled, and the session was used to get to know the participant and make him feel at ease. In some instances, interviews were conducted in the presence of a family member or spouse, as requested by the participant. After the interviews, the researcher spent a few minutes answering any further questions the participant had.

### Data analysis

Interviews from all participants were audio recorded using a voice recording device and transcribed verbatim by the interviewer (lead author of this paper). The thematic analysis technique outlined by Guest et al. (2012) was used to analyze the data. Thematic analysis was appropriate for this study as it revealed the shared experiences of the participants through the recurring themes found in the data. In addition, the NVivo 12 qualitative analysis software was used to assist in organizing the data and identifying the themes. The data analysis was conducted in the following steps:

1. Each of the participants’ transcripts was read and re-read in an interpretative manner to gain a better understanding of each participant’s experience.
2. After becoming more familiar with the transcripts, coding of the transcript began by identifying key segments of the transcripts that provided details that addressed the research questions. Open coding was used, meaning that there were no predetermined codes; each code was established based on what was deemed as meaningful information that addressed the aims of the study and the research questions.
3. Following this, the search for themes began. This involved identifying key concepts and patterns that emerged within each transcript and assigning comments to each of the meaning units identified. These themes were arranged in the order that they emerged during the analysis.
4. After identifying the themes, each theme was reviewed. This aimed at determining whether themes made sense, did not overlap, were supported by the transcripts, and addressed the research questions.
5. Each theme was named and defined based on what emerged on the careful reading of the transcript.
6. During data analysis, it became evident that data saturation was reached at 7 participants, i.e., no new themes emerged from the transcripts of participants 8, 9 and 10. At this stage, the three themes discussed in the results section had emerged.
7. Once all transcripts had been analyzed and the final themes had been identified, the write-up of the results was conducted. This stage involved providing a narrative account of the themes that emerged from the transcripts by using an overarching comment for each theme and providing direct quotes from the transcripts to illustrate and provide evidence for it.

### Ethical considerations

To ensure validity and reliability, participation was voluntary and was secured by ensuring signed consent forms from all participants. Participants were assured that the information they provided during the interviews would be kept anonymous, with their identities being protected by using pseudonyms and conducting interviews privately and one on one (unless participants requested that a family member present). All interviews were transcribed verbatim and a systematic method for identifying, analyzing, and organizing data into discrete categories that are comparable and replicable was used [Guest et al. (2012) thematic analysis]. All findings were presented in an authentic manner in all research outputs and all participants’ responses were represented in the results. Electronic and printed formats of the information gathered during the study were stored in password-protected files and secure files to secure the coded information.

### Reflexivity

Given that one of the core impairments in autism is related to social engagement and reciprocity (APA, 2013), it was recognized that the act of engaging in interviews might be challenging for participants. As noted previously under Data Collection, the interviewer role-played the interview with an NDC counsellor. This process enabled the interviewer to become more sensitive and moderate her style of engagement to maximize the quality of the interviews, a roleplay of the interview with a clinician from the NDC, specializing in autism, was conducted. Given that the history of South Africa is characterized by racism and the power differences that still exist, the participants’ perceptions of the interviewer as a young black isiXhosa speaking woman interviewing older white, predominantly Afrikaans speaking men may have limited the extent to which the participants were willing to discuss intimate details of their experiences. In addition, the interviewer’s tendency to be overly cautious, thereby avoiding further probing of responses given by participants that contained sensitive information (that may or may not have been in line with the objectives of the study), may have limited the richness of the data gathered. Notably, although majority of the participants were Afrikaans speaking, only one participant experienced challenges expressing himself in English. To mitigate this, the participant’s wife translated throughout the interview. Lastly, it was noted that the family members of the participants were very aware of the participants challenges in social interactions and were cautious as to who the participants engaged with, including the interviewer. However, when they became aware of the interviewer’s awareness of the challenges ASD may present in the interview for the participants, they appeared more at ease with the participants engaging in the interviews.

## Results

### Participant demographics

To contextualize the results and allow for an understanding of the life circumstances of the participants who engaged in the study, Table 1 is presented below.

**Table 1:** Table representing the demographical information of the participants

Participant’s Pseudonym	Age at time of interview		Age at time of diagnosis		Year of diagnosis	Marital/ partnership status	Number and age of children	Living arrangements	Highest level of education		Qualifications		Employment status
Albert	35		32		2015	Single	None	Lives in parent’s flat who assist him with minor housework and transport	Bachelor’s Degree		Bachelor of Science: Computing		Unemployed, student
Anthony	28		26		2016	Single	None	Lives with his parents and is dependent on his parents as he is unemployed. He also depends on his friends for things like transportation	Information not available		Information not available		Unemployed
Boris	47		47		April 2018	Married	Children: 1; Ages: 9	Lives with his wife and child, no assistance required/ received	Information not available		Information not available		Selfemployed
Francois	41		40		Feb 2018	Married	Children: 3; Ages: 2, 7 and 9	Lives with his wife and kids in their own home, no assistance required/ received	National Diploma		National Diploma: Information Technology		Employed
Hennie	35		33		2016	Married	No children	Lives with his wife, and receives advice from his psychologist and wife on how to deal with people	Project Management NQF8. MBA Essentials NQF8		I do not have a degree but have a lot of certificates. I am planning to do my BCOM – Financial Management		Employed
Hugo	44		40		2017	Married	Children: 2; Aged: 16 and 19	Lives with his wife and children, no assistance required/ received	Technicon qualification		Technicon qualification		Employed
Luke		39		35	2014	Single	None	Lives alone, and receives financial assistance from his mother	National Diploma		National Diploma: Information Technology		Employed
Nicholas		56		56	2018	Married	Children: 3; Ages: 23, 14 and 8	Lives with his wife and children, no assistance required/ received	Honours degree		Undergraduate degree: Marketing Management Honours degree: Business		Selfemployed
Mendel		48		44	2014	Married	Children: 4; Ages:	Lives with his wife and children, and gets assistance from his wife	Grade 12		High School certificate		Self-employed
Philip		64	64		February 2018	Married	Children: 1; Age/s: 18	Lives with his wife and son. Don’t receive or need assistance	Graduate Diploma, Post Graduate Certificate and MSc	Post Degree in Engineering, Media, Film & Television, Marketing and Humanities equivalent		Unemployed	

## Demographic information related to participants

The three main themes that emerged through the analysis were, failure to diagnose ASD in childhood despite signs and symptoms, ramifications of missed / misdiagnosis in childhood and adulthood on psychological well-being, and the impact of receiving a diagnosis of ASD in adulthood.

### Failure to diagnose ASD in childhood despite signs and symptoms

All participants in the present study reported presenting with were identified as challenging behaviours throughout their childhoods, with parents and teachers perceiving them as being no ordinary child. These challenges were often discounted as just “boys being boys” or were understood as being the result of other mental health conditions. The underlying cause of these challenges, being ASD, appeared to be overlooked, misdiagnosed, or misinterpreted. The difficulties reportedly experienced by participants in their early childhood development included behavioural (temper tantrums and restlessness) and social (being extremely shy and withdrawn and being perceived as being socially inappropriate) challenges as well as regression in development (such as when a child who had developed age-appropriate speech began to lose the skills around the age of five to eight years). Although those around them (family and teachers) perceived them as being “no ordinary child”, their difficulties were often not addressed, and ASD was not considered as being the core cause of their challenges.

“I was a hyperactive child, difficult. I was way more difficult than my brother and sister who followed me, many times. Um, I was difficult at school… the standard sort of report would be I’m a disruptive influence and it’s not fair to the children who, who want to, uh, want to learn in the class. So that was a common thread through, through my education. I was disruptive” (Nicholas)

“If someone falls off their bike, I'll start laughing because I think it's funny. Um, other people will be like shocked, like geez, how can you laugh at that, while to me it's funny” (Boris)

“When I was a kid, I would say stuff to people without thinking what I was saying, sometimes hurting people's feelings because I would say something negative without realizing it” (Francois)

“… not really giving people a chance to speak because I'll be getting into whatever I was talking about” (Anthony)

“I mean I started stuttering at eight, but they never really took me for counselling or helping or anything like that.” (Luke)

Participants understood the oversight of their core difficulties of being on the spectrum in several different ways. One participant reported their difficulties being normalized based on gender stereotypes ascribed to boy children.

“My sister works with children with autism and when I told her she said that there is no way I have autism she knows what autism looks like and I don’t have it so I must stop looking for attention. All men are like that if that’s the case then all men should have autism” (Philip)

Five others reported a tendency of those around them minimizing their difficulties as not being a significant problem.

“They kind of just, I don't want it to sound rude, but maybe they turned a blind eye or I'm not quite sure but if they were concerned, they didn't show it.” (Luke)

“I did well at school, and I had a few friends and stuff like that. So, they were happy with me as a child growing up.” (Francois)

The inaction of addressing the above-mentioned concerns was attributed to various reasons, such as lack of knowledge around the disorder, lack of support services, passivity and personal challenges primary caregivers were experiencing (including struggling from their own undiagnosed ASD).

“I mean my father took very little notice of me, possibly because he had the same condition” (Philip)

“We have a sneaky suspicion that my mom might be autistic as well.” (Albertus)

“Around about five-ish or so if I'm not mistaken…There was a strong suspicion that I could have been autistic um… my father was also a [professional]… remember in the past there wasn't really an emphasis on the whole subject so they, they didn't really go into autism and in the past, they will just say there's something wrong with you sort yourself out… unfortunately, you know I think these days a lot of people, I know somebody personally whose son is also on the spectrum and they do a lot more for the children these days” (Hennie)

Teachers were also regarded as professionals whose knowledge of conditions on the autism spectrum was limited resulting in children being labelled as “difficult” or “shy”.

“I also had a lot of anxiety and that caused me to find it hard to express myself in class. I would literally turn red in the face when the teacher asked me a question to the extent that the teacher wouldn’t ask other kids questions and would joke to the whole class and say: “Let’s ask Philip a question and see how red he will get.” That made me even more vulnerable to bullying.” (Philip)

Three participants described their families as being accepting of their uniqueness and did not perceive it as problematic, which also led to not seeking professional help for their autism-related presentations.

“My dad would frequently ask me things like what is wrong and what not because you know, my normal face, is not a smiling happy face… So, um, I think, I don’t think there was much concern about it like I said, my family’s really fairly accepting. They don’t really push me out of my comfort zone” (Anthony)

However, this was experienced by participants as problematic as it contributed to parents not seeking professional help and getting a diagnosis during their early childhood. Participants also felt that their difficulties were not addressed and may have been worsened by not gaining access to appropriate support services.

“… a lot of the things I'm having to learn in terms of like socializing stuff and picking up social cues and stuff, it's so difficult now as an adult with a lot of other worries and other things to take note of.” (Anthony)

The missed/ misdiagnosis for six of the participants, had psychological sequalae with increased distress resulting in depression and anxiety. Participants were then treated for these mental health illnesses; however, no interventions for autism were provided.

“I got diagnosed with generalized anxiety and then I went to the psychiatrist for three, four, five years and then he started diagnosing me with OCD and I said, wait a minute, that doesn't fit” (Albert)

“Over a period of years, I've always figured out that I’m different I, I, I sense things differently and see things differently and so, I mean I've always known there is some kind of variation, so basically they just said it's just depression and so they, they, they are treating depression, but nobody was aware of, of, of the Asperger's” (Boris)

The diagnosis in adulthood appeared to be the result of two scenarios. The first was that participants only received a diagnosis of autism through seeking help for their children’s difficulties, only to discover that they also had a condition on the autism spectrum. In addition, participants indicated that they had seen several health professionals regarding their children’s difficulties without any success and only received a successful referral when someone, who knew an individual diagnosed with autism:

“My daughter was 18 at that stage and we've been through psychiatry, psychologist, everything because we had problems with her, she, she didn’t cope in the normal or function well in normal circumstances…And then somebody told us that her brother was diagnosed with Asperger’s and that [therapist name] was the leader in the field… then we took my daughter there and we had the first session and he sat and he talked and while we're talking, everything that he said fell into place... I knew that was exactly as he was describing it. And then the next moment he turned to my partner, he said, but he wants to ask a few questions about me, and he asked the questions and as he was asking it, I just started smiling because I knew, I knew that what it means…he called us back and he said, listen…he wants to see me the next, the next week. So, then I waited for my session and then it was discovered.” (Hugo)

Alternatively, ongoing difficulties leading to a crisis propelled participants to seek a diagnosis. Given the growing recognition of neurodivergence, including milder symptoms of ASD, and the impact of mild/ “high functioning” forms of ASD, there were more resources to draw on and an increased likelihood of being diagnosed as being on the spectrum.

“And I think it was through a struggle of that and was a lot of strain on our marriage and everything. It was really a tipping point or breaking point. It just sort of dawned on me because my awareness of autism or Asperger’s had improved, it had increased a lot during this time. So, it dawned on me, I thought maybe I’ve got that, you know... And then I phoned [therapist name], and almost on the phone I knew” (Nicholas)

“I worked at the company, and everything got a lot. We were moving from one place to another and there was a lot of stress at work and everything got overwhelming and as you would say I got a meltdown and then we said no we have to make a plan this can't carry on…I struggle to deal with crises and things like that… my wife is also a psychometrist in psychology…she did a bit of reading up on the subject and realized that there might be a form of autism in me… that's when I was diagnosed by [mention’s therapist’s name] and his team” (Hennie)

Nonetheless, the route to a diagnosis to ASD could still sometimes be circuitous. For example, in the case of Anthony, there is a recognition of neurodivergence identified as ADHD, however, only later was he recognized as being on the spectrum.

“I was diagnosed with ADHD… At first, I was put on Ritalin, eventually I went off it because I found myself getting into, you know, I'll sit there and people would talk to me and I wouldn’t um, you know, I don't know, I wouldn't register the fact that they're talking to me because I was too focused on whatever I was doing” (Anthony)

These findings suggest that early indicators of autism (such as challenging behaviour, being socially inappropriate/ withdrawn and being regarded as being “no ordinary child”) were evident during participants’ childhoods. However, these indicators were often missed, attributed to other causes outside of ASD or normalized. Participants understood their challenges being overlooked/ not being addressed as being a result of lack of knowledge (on the part of parents and/or professionals) leading to a lack of support as well as mistaking the symptoms as being attributable to other mental illnesses. Of note, the lack of early diagnosis of ASD and gaining access to appropriate support services had significant psychological sequela for participants. For most participants, a diagnosis was only gained during times of crisis, often when increased adulthood demands exceeded their abilities to cope during to ASD related challenges.

### Ramifications of missed /misdiagnose in childhood and adulthood on psychological well-being

All participants expressed that a missed or misdiagnosis and being diagnosed in adulthood had a significant impact on their lives. The lack of understanding of the underlying cause of their behavioural challenges led to ineffective measures, such as discipline, being used as a strategy to correct the “difficulties”. In addition, the lack of knowledge about their challenges caused distress and left some participants vulnerable to being bullied and ostracized. These feelings of isolation and lack of insight into their difficulties, further led to emotional and psychological distress with two participants reporting suicidal ideations.

“Well, the way to address those concerns in those days would be discipline” (Nicholas)

“I had difficulties making friends and as a result, I was severely bullied. I’ve been cut on my back with a razor, I’ve had my head pushed into a toilet, I’ve been physically beaten. I experienced bullying at school, and I also experienced bullying at home from my father and all that made things even more difficult to bear… And I had some suicidal tendencies, and I had some just general frustrations because I couldn't quite understand what was happening inside. I felt isolated and anxious and felt like I couldn’t take it anymore.” (Philip)

“No parent should have to bury their child. I was going to kill myself after my parents had died” (Luke)

The challenges extended beyond their social world, also affecting their academic work leaving them to navigate through their schooling career without adequate support.

“My biggest challenge is a lack of a lack of concentration. So, so I jump from here to there to there” (Hugo)

“I struggle with like writing neatly and quickly. So basically, what I did is I like sat in class and ignore the teacher and slept and read a book or something then I did the work myself at home and then that's how I passed” (Albertus).

It was also evident that the lack of understanding, on the part of parents, professionals, and the public, regarding participants’ challenges hindered them from obtaining and retaining employment, which played an integral role for them in maintaining ‘normalcy’. Without understanding their challenges, participants reported being disadvantaged, as they had no way of explaining or defending their idiosyncrasies, such as not being able to follow instructions and meeting deadlines, which led to participants losing their jobs. This negatively impacted them because, without employment, they could not be independent.

“If they tell me you have to do it like this, I wanted to know why I can’t do it this way because this is easier for me… after three years they couldn't handle me anymore. They get the union in, and the union told me that I have to listen to the boss and that is the way that it should be and if I'm not listening, they can fire me.” (Mendel)

“I can't just walk in and start a job. I need time to climatize and get used to all the people and things like that… also going into the interviews, I just couldn't deal with interviews” (Luke)

“I haven't been able to keep a job… I worked there for about three months now this was before my diagnosis, um, and they couldn't understand why I wouldn't, you know if I was struggling with something, why I wouldn't communicate with them… on top of, on top of that, I started getting tired after a while so I can keep, I can keep a job going for a couple of weeks or months… And that's what happened with [mentions company name] um, eventually they let me go. They used the probationary period to let me go, at the end of the probation they let me go, um, because I couldn't quite understand why I wasn't doing so well why I wasn't communicating when something went wrong or anything like that. It might have been different say if I had my diagnosis at that point though, I think they would have understood a lot better” (Anthony)

“If you ever meet someone and they ask, you know, what do you do for a living and where do you live? I was so embarrassed to say I don't have a job and I live with my mom.” (Luke)

In some instances, participants managed these difficulties by finding jobs that would allow them to obtain some sort of normalcy, such as working in environments where they would not have to engage with others, which minimized the likelihood that they would have any miscommunication with others. On the other hand, three of the participants described their autism-related traits as being an advantage within the workplace allowing them to thrive.

“At school, I was always good at accounting and math… I switched over to BSC mathematics … If you’re focusing on writing software, as soon as you have a break in concentration, it actually takes you more time to get back into that zone before that…. my job allows me to function on my own most of the time… working here, the environment allows me to function more normally” (Francois)

“There was a definite structure. There were rules to the game. Those rules of engagement that I could actually pick up quite quickly and to not get emotionally involved in stuff and tied into that web is, it is a plus... Business was like… I understood the rules of engagement. I understood when I was interested in it…I learned, and I was successful” (Nicholas)

Participants described trying to manage their undiagnosed daily challenges and difficulties with a range of strategies. Some of the coping strategies employed were apparently more adaptive, like masking. It is important to highlight that amongst these participants, masking was two folded. Firstly, some participants engaged in masking as an adaptive way to navigate through their social environment. For example, following career paths where they could use their professional image and duties to make their challenges less “obvious”. This can be regarded as an adaptive use of camouflaging of autism-related challenges as participants did not describe being distressed by it.

“I can be very charming. I can be well spoken. Uh, I think those are the, probably the main, the main factors or the main tools… I would imagine, it’s like acting... you watch movies, maybe you watch documentaries, you watch whatever. You take bits and pieces and you put them together.” (Boris)

“Even the role I played at work allowed me to put on an act because you would go to a pub after work to network with potential clients and you would sit and recite a script to them that tells them what you do and how it will be beneficial to them. So, in those settings, my difficulties were not that obvious.” (Philip)

On the other hand, participants who described using masking to “hide” their challenges found it to be exhausting and distressing, drawing attention to the fact that masking autism-related traits can be both adaptive and maladaptive.

“My whole life has been a strategy on hiding it, mainly through reading novels about characters and watching films, watching tv shows, to try and get a sense from those, how people behave in certain situations and adopting some of those characteristics to be my armor, if you'd like it, to protect me from autism.” (Philip)

“I become obsessed with movies because they helped me make sense of things and taught me how to act in certain situations. It is very exhausting.” (Philip)

“My sense of identity was wrapped up in this, in this role I grew into... So, you can do that by being a successful businessman and you can also do that by living the lifestyle that Mick Jagger. It's only when, it’s only when you can’t go any further with those two ways of living that you have some sort of death, some symbolic death. Yeah. And then through that does some sort of search begins for yourself” (Nicholas)

Other strategies aimed at managing participants daily undiagnosed challenges were arguably maladaptive, such as substance use and high-risk taking behaviour.

“I think in my coping mechanisms, they have been a cost like alcohol, drugs, sex and women, you know, all these types of things” (Nicholas)

Without an adequate understanding of their challenges and the necessary skills training and support, it appears that participants were left with very limited options.

Overall, the late diagnosis of ASD had significant ramifications for participants in this study as they were left to navigate their daily autism related challenges without understanding them. These challenges extended beyond participants’ social world, affecting their academic performance, career prospects and ability to live independently. This had a profound impact on participants’ psychological well-being, to the extent of participants experiencing suicidal ideation. Of note, without an understanding of their challenges and accessing appropriate support services, participants employed maladaptive coping strategies (such as masking, substance use and engaging in risky behaviour) as an attempt to manage their challenges.

### The impact of receiving a diagnosis of ASD in adulthood

Nine of the participants were finally diagnosed with ASD between the ages of 30 and 50. Seven participants reported that receiving a diagnosis during childhood may have positively changed their trajectory and life outcomes. They expressed feelings of loss in terms of all the support and interventions they could have received during childhood that are no longer available or are not as effective during adulthood. While they expressed deep feelings of regret and loss, they remained hopeful as they began to understand their challenges and how they could best manage them.

“I mean, education would have been different. Schooling would have been different. Just, everything would have been different, you know, and not forced” (Boris)

“I mean I've missed so many milestones in my life. I mean I'm almost 40 now and I'm stuck maybe in my twenties. You know what I mean? I've missed out so much on life” (Luke)

“I cried. And then I felt very frustrated and angry about all the hard work I’d put into psychotherapy, etc. In London, the amount of money I’d spent on, the time I spent on it when clearly, they were barking up the wrong tree with me...but also, I felt very relieved that I'm hopeful, finally somebody has told me, actually, I'm suffering from a condition that I could do nothing about” (Philip)

“Working with psychologists on how to deal with situations and getting the necessary training makes the future look more brighter… I work a lot with my psychologist and psychiatrist to give me the guidelines on how to work with or how to deal with situations” (Hennie)

Generally, participants reported that gaining an understanding of their challenges allowed them to find ways of managing them and to be more accepting of who they are.

“It has helped me understand why I find it difficult building connections with people. I struggled with certain things, especially with a PDA [Pathological Demand Avoidance] diagnosis, it helped me to understand like, why I struggled to get things done, why it takes me a lot longer to get things done… so yeah, it has, it has helped me understand all those things in the past and has given me fuel for future actions” (Anthony)

“There’s more accepting of who I am…. You don’t try and copy other people and try and fit in. You’re trying to be yourself… It’s almost every time it's like you're wearing a mask, or you play a character around people. But I've kind of stopped trying to do that” (Luke)

“Finding that out, is a huge gift… I was trying to piece myself together, in a way, understand myself, but it was like I was putting a jigsaw together that was upside down, you know, the cardboard that was facing up. And then when I got that one piece, it was like the centerpiece. And it was the first time there was actually a picture and I put that piece in and all of a sudden you flipped the whole thing over”. (Nicholas)

In instances where participants had children who also had conditions on the autism spectrum, they described how the knowledge and understanding of their diagnosis helped them understand their children’s difficulties and how they could best manage and support them. They further reported their families’ acceptance of them, before and after their diagnosis, allows them to be their authentic self.

“I’ve learned to be more patient with my daughter… you see things differently and you understand more. It’s a little bit clearer now, you know, why would you say, you know, why do you do the things you do? It is because of that. So, you understand now, you, you put things into context.” (Boris)

“My family's very accepting… Um, I mean my family for a start have been a big help. I think if they were less supportive of it, I know some parents with Aspie or autistic children are either not invested or they almost against the diagnosis itself and I think that can make a lot more difficult, but I’m fortunate enough that a lot of my friends tend to and my family as well they’re very accepting, they don't they don't perceive it as anything wrong. And because of that acceptance, it kind of made it easier for me to accept myself” (Anthony)

One participant expressed being happy about receiving the diagnosis in adulthood as they felt they wouldn’t want to alter the course of their lives, two participants felt indifferent stating that being diagnosed with autism has not influenced how they perceive themselves. Additionally, two participants felt inclined to distinguish between self and diagnosis to avoid labels and possibly stereotyping, some emphasized and took pride in the positive traits they have because of autism.

“I'm glad I didn't because I wouldn't want to change my life… I developed coping, ways to cope with them.” (Nicholas)

“I'm not really going to change who I am. I’ve, I've adopted and adapted my life through the years to fit in more socially...” (Francois)

“But it’s kind of difficult because like, I’m actually at a bit of a phase at the moment when I'm trying to actually work out how much of this, of what I'm experiencing is, is uh, is out of my control as a result of Asperger's or autism and how much of it is within my control because I don't really want to fall into the trap of explaining to people, oh, I'm not doing this because I'm autistic and using it as a crutch or an excuse… I kind of fear that people will see me differently if I start to bring it up and that they might have to act in a certain way when they're dealing with me. So, I try and avoid that” (Anthony)

“I’ll tell them like I'm like Sheldon Cooper from Big Bang and everyone is like yeah that’s a cool guy, yeah there’s another character House from the series on the autism spectrum… I actually use it as a bragging feature these days” (Hennie)

Although receiving a diagnosis appeared to have helped participants to understand their challenges and develop effective ways of managing them, participants reported feeling as though the future will not be any different from the past, as they were given the diagnosis as there are limited services providing support for adults on the autism spectrum.

“I find it is difficult finding services for adults with autism. I have contacted people who work with children with autism 2 or 3 times before, and they could not refer me to any services for adults. I finally found some services through my psychologist, who actually knew the person personally.” (Albertus)

Notably, following receiving a diagnosis, those who chose to disclose to their employers described having advantages such as being accommodated where necessary, while others chose not to disclose for fear of being discriminated against.

“… it’s the first company that I’ve actually disclosed that I’m on the autism spectrum. I even told the HR about it… they've been very accommodating in the workspace. So, they know how to work with me… My work colleagues also know about it so we've now built-in measures to accommodate for my needs” (Hennie)

“I know a lot of jobs and businesses; they wouldn't hire you if they find out that you are on the spectrum.” (Anthony)

The main finding of this theme was that participants perceived that receiving a diagnosis of autism early in their childhood may have had a significant positive impact in their life trajectory and life outcomes. Following receiving a diagnosis during their adulthood, participants expressed that it has helped them with greater self-acceptance and has increased their understanding of their ASD related challenges and how to manage them. Notably, participants’ self-acceptance was largely influenced by their families’ acceptance of them and their diagnosis. Participants who were more self-accepting, were also more likely to disclose their diagnosis to their employers, which they found to be helpful as their challenges were better understood and accommodations were made available where necessary. There were also instances where participants chose to distinguish themselves from the ASD diagnosis due to the perceived stigma attached to the diagnosis. These participants self-reported being reluctant to disclose their diagnosis to their employers due to concerns of being stigmatized and/ or discriminated against.

## Discussion

In keeping with the United States Centers for Disease Control and Prevention’s (2009) report that indicators of autism are evident before the age of five, in this study, it was found that participants presented with initial symptoms that were indicative of ASD within the first five years of life. However, these symptoms were either missed or misdiagnosed. All participants described exhibiting social impairments, loss of language, and/ or challenging behaviour within the early stages of development. Interestingly, social impairment was the most prominent early indicator of autism that participants identified with. The symptoms described by the participants in this study are in line with the findings of other studies which describe such difficulties as being early concerns of parents of children who were later diagnosed with autism (De Giacomo & Fombonne, 1998; Welton, 2004; Young et al., 2003; Zeleke et al., 2017). This suggests that some of the delays in identifying early symptomatic features described by the participants may reflect parents’ and professionals’ difficulty in understanding them, rather than the absence of symptoms per se. The experience of the participants in this study also points to the complexity of symptomatology associated with ASD because in some cases there are periods of apparent typical development followed by loss of skills that were already developed.

Participants identified several factors which contributed to symptoms being overlooked or misdiagnosed, including lack of awareness about the condition, personal challenges of caregivers, and normalization of symptoms due to gender stereotypes. Furthermore, the participants being high functioning resulted in them being reframed through normalizing their ASD related symptoms. This misperception or lack of an understanding is not unusual and has been reported in a number of other studies (e.g., Aggarwal & Angus, 2015; Kishore & Basu, 2011; Mandell et al., 2009; Zeleke et al., 2017). In addition to symptoms being misperceived or not understood by parents, participants also reported a lack of knowledge by professionals who were consulted, including health professionals and teachers. This was not surprising, given that there has been an exponential growth in the diagnosis of ASD in the last 10 to 15 years, prior to which there was limited research and understanding of the presentation of milder presentations or high functioning ASD (Franz et al., 2017; Silberman, 2015; Zeleke et al., 2017). Lastly, it was also not surprising that participants in this study attributed factors, such as gender norms of ASD symptoms, as contributing to their late diagnosis, as this has also been observed in other studies (Baron-Cohen et al., 2005).

It was also found that when professional help was sought, participants’ difficulties were misunderstood and were not considered as being symptomatic of ASD. This had a detrimental effect on the participants as it led to receiving multiple misdiagnoses (with the common misdiagnoses being ADHD, depression, and anxiety) and being administered various treatment methods which were mostly ineffective and, in some cases, resulted in severe side effects. This was in line with Bargiela et al.’s (2016) finding that females who were diagnosed with ASD in their adulthood had received similar misdiagnoses, and, in some cases, no diagnosis was given at all. This tendency to misdiagnose individuals with autism has been linked to its co-morbidity with other conditions, such as ADHD and anxiety (Davidocitch et al., 2015; Hendrickx, 2015; Hurlbutt & Chalmers, 2002; Leyfer et al., 2006). In addition, prior to the development in understanding and assessment for ASD evident over the past 15 years, it is likely that professionals lacked training in screening for ASD and had limited diagnostic tools for assessment thereof. This may have contributed to the why the participants in this study were misdiagnosed and/or whose diagnosis was missed (Abubakar et al., 2016; NICE, 2012). The lack of an early diagnosis was also found to have impacted participants’ emotional and psychological wellbeing. Of note, several participants were finally diagnosed during times a crisis when their ASD related challenges significantly affected their ability to meet the demands of being a self-sufficient and productive adult member of society.

The late diagnosis of autism was found to have significant ramifications for participants in this study. The lack of understanding of their ASD related challenges resulted in participants being isolated, ostracized and bullied throughout their childhood into adulthood. This significantly affected their emotional and psychological well-being, with two participants reporting having suicidal ideation. Similarly, Leyfer et al. (2006) states that individuals who are diagnosed late, have been found to be at high risk of depression, anxiety, and suicide. It was also found that receiving a late diagnosis affected participants’ ability of attaining a sense of normalcy, as their ASD related challenges affected their ability to progress academically, as well as their ability to attain and retain employment. This is in line with DePape and Lindsay (2016), Hendrickx (2009, 2015), and Barnard et al. (2001) who report that adults with autism experience difficulties in social outcomes, obtaining and maintaining employment, and they continue to be dependent on their family, relying on them for support. Of note, it was found that ineffective strategies of managing participants’ ASD related challenges were used by caregivers and participants. Participants in this study described using maladaptive coping strategies such as masking, substance use and withdrawn behaviour, while caregivers employed strategies such as discipline as an attempt to manage challenging behaviour. This is in keeping with DePape and Lindsay’s (2016) findings that the anxiety induced by feeling different from others resulted in maladaptive behaviours, like relying on alcohol and social withdrawal as well as masking as described by Bargiela et al. (2016).

The phenomenon of masking social difficulties was described by participants as a deliberate attempt to learn and use neurotypical social skills to fit in. Several participants described engaging in masking at an unconscious level, through observing the behaviors of others and mimicking it without being aware of it. Masking had positive outcomes, in that it provided a way for participants to manage their difficulties and navigate their way through the world. However, masking also resulted in negative outcomes, because the effort required to hide challenges and differences resulted in exhaustion and an increased sense of isolation and distress at not being able to fit in. While masking is typically seen in females (Bargiela et al., 2016; Hiller et al., 2015), the findings from this study suggest that males may also engage in masking their difficulties and may suggest a need for caution when making such gendered assertions.

Overall, participants in this study expressed that receiving a diagnosis had significant positive benefits for them, as a better understanding of their challenges allowed them to develop tools that will help them move forward. They expressed how they now understand the underlying cause for their difficulties in forming relationships and other challenges they had, but most importantly, how gaining this understanding has enabled them to be more accepting of who they are. Hurlbutt and Chalmers (2002), have noted that the level of acceptance that individuals with autism have of their diagnosis is influenced by whether family and friends are accepting of the diagnosis. In this study, it was found that participants whose families and friends were accepting, and supportive following disclosure of the diagnosis resulted in participants themselves being more accepting of their diagnosis and finding adaptive ways to manage some of their challenges, including disclosure in the workplace, which then allowed for requests for accommodations. In contrast, participants who chose not to disclose their diagnosis to their employers due to various reasons, such as the possibility of being labelled and the stigma attached to conditions on the autism spectrum, did not express the same level of acceptance of their diagnosis as those who had disclosed and received support from their families and friends.

The results of this study also point to how receiving a diagnosis earlier may have made a significant positive impact on participants’ experiences and life outcomes. It has been suggested that social skills, which are impaired in individuals with autism can be enhanced and developed, especially through intervention at a young age (Aldred et al., 2008). Early interventions have been found to reduce developmental gaps that individuals with ASD experience compared to neurotypical individuals (Rogers et al., 2014). A late diagnosis means that a critical period was missed in which interventions could be introduced to scaffold and support the development of skills and strategies which could mitigate the distress and challenges experienced later in life (Hendrickx, 2015; Koegel et al., 2014). Similar findings were evident in this study as participants expressed a sense of loss, due to receiving a late diagnosis, as they did not receive support to help them manage their challenges, which they believed would have positively influenced their life trajectories and outcomes. Furthermore, receiving a late diagnosis left them feeling helpless as there are limited support services available for adults with autism, which is in line with what is reported by Lewis (2016).

### Implications of findings

The key findings of this study suggest that a late diagnosis of ASD has significant negative ramifications. Late diagnosis negatively impacts on individuals’ psychological well-being (due to factors such as social isolation, bullying and psychological distress caused by lack of understanding of ASD related difficulties). Individuals are also impacted in that, without support services, they become restricted in their abilities to thrive as there is not support for and/ or within families and schools to assist with scaffolding the environment to help individuals with ASD better manage their challenges. The clinical implication for these findings emphasizes the urgent need for teachers and mental health professionals to be trained to better screen, assess and diagnose milder presentations of ASD as early as possible. Furthermore, parents need to be provided psychoeducation to raise awareness for the disorder and to access available support services.

### Strengths and limitations

As a preliminary in-depth study, the sample size was small, and therefore, cannot assume broader generalization. However, the findings can be regarded as a stepping-stone to a larger quantitative study investigating the above-mentioned factors in a more representative sample, possibly comparing males and females. The study also consisted of participants who were white middle-class males, because the NDC is a private facility and thus offers services to individuals with their own financial means or medical aid. This is a minority of South Africans, as, due to economic constraints, most of the population relies on public health services (Statistics South Africa, 2017). The data collection process itself may have limited the number of participants willing and able to take part since difficulties in social interaction is a core challenge in individuals with ASD. A less anxiety provoking approach to data collection may have been the use of questionnaires, with the researcher further sending follow-up questions to each participant for further probing in areas that were relating to the study’s objectives. With that being said, the use of interviews provided an opportunity to gain an in-depth understanding of the participants’ experiences and participants were eager to share these with the researcher and valued research focused on their experiences. A key strength of this study is that in contributes to an area of research in which there is a limited body of knowledge. A total of five studies on individuals who received a late diagnosis of ASD was found, only one of which was focused on males. Of the five studies all have been conducted in the global north and no studies conducted in Sub-Saharan Africa were found.

## Conclusion, recommendations and future direction

The purpose of this study was to explore the experiences of males who were diagnosed in adulthood with ASD. Through in-depth qualitative interviews the research sought to explore and understand symptoms and challenges in childhood associated with ASD which were missed or misdiagnosed, the ramifications thereof, and the impact of receiving a diagnosis of ASD in adulthood. It was found that indicators of autism were evident during early and later childhood. However, these symptoms were either overlooked and/or normalized by parents and teachers or understood as due to a psychiatric disorder other than ASD by mental health practitioners. This had a significant impact on the overall life experiences and outcomes for the participants in this study, as they were forced to navigate through their lives without an understanding their difficulties. In addition, as these individuals progressed through life, they encountered numerous challenges and adopted various strategies to cope with some of their autism-related difficulties. Some of these coping mechanisms were maladaptive, while those that were considered as being adaptive, had unintended negative consequences. Furthermore, due to limited availability and/or access to support services for adults with autism, participants felt that the future, following their diagnosis, does not look any brighter than the past.

These findings suggest several research paths going forward. Future research should further explore the impact of late diagnosis of autism using a larger sample size, possibly comparing males and females, especially with reference to masking and whether there are gender differences. Future research should also focus on exploring the experiences of parents of individuals diagnosed with autism in adulthood, focusing on investigating their understanding (before and after their children’s diagnosis) and whether some of the ASD related challenges resonate with them (especially considering that participants suspected that their parents may be on the autism spectrum). There is also an urgent need for future research to investigate the personal and professional support needed by individuals diagnosed with autism during adulthood and how these needs can be met. Future research should also explore the understanding that professionals (such as doctors, teachers, and mental health practitioners) who engage with children have regarding autism and how likely they are to screen for ASD when children present with social, behavioural and other developmental challenges. Lastly, research focused on understanding the impact that socio-economic status has on the likelihood of individuals receiving an ASD diagnosis is paramount. This question is even more pertinent in emerging economies such as those in Sub-Saharan Africa where access to resources is significantly challenging.

## Data Availability

The datasets generated during and/or analyzed during the current study are available from the corresponding author on reasonable request.

## Footnotes

1 High functioning autism is not an official diagnosis, but it is used to describe the level of ability of an individual with an autism spectrum disorder (Borgman, 2015), typically describing individuals who to a large extent ‘blend in’ with neurotypicals. There has been controversy around the term, with arguments centred around the fact that the term ‘functioning’ is vaguely defined and leads to misunderstanding (Borgman, 2015; Robison, 2014). For the purpose of this study, the term high functioning will be used to refer to individuals who were diagnosed with Asperger’s and/ or individuals who were diagnosed with ASD and are classified as presenting with ‘milder symptoms’ (that is, aren’t severely impacted in their social communication abilities) of autism and are able to verbally express themselves and their experiences.

2 For the purpose of this study, the terms delayed, and/or late diagnosis will be used interchangeably to refer to an adulthood diagnosis of autism that occurs after the South African legal age of 18 (Legal Aid South Africa 2015).
